# Exclusively Pumping Women’s Milk Production and Pumping and Feeding Dynamics—An Online Survey

**DOI:** 10.3390/healthcare14152436

**Published:** 2026-08-06

**Authors:** Zoya Gridneva, Jacki L. McEachran, Demelza J. Ireland, Sharon L. Perrella, Donna T. Geddes

**Affiliations:** 1 School of Molecular Sciences, The University of Western Australia, Crawley, WA 6009, Australia; jacki.mceachran@uwa.edu.au (J.L.M.); sharon.perrella@uwa.edu.au (S.L.P.); donna.geddes@uwa.edu.au (D.T.G.); 2 ABREAST Network, Perth, WA 6000, Australia; 3 UWA Centre for Human Lactation Research and Translation, The University of Western Australia, Crawley, WA 6009, Australia; 4 School of Biomedical Sciences, The University of Western Australia, Crawley, WA 6009, Australia; demelza.ireland@uwa.edu.au

**Keywords:** milk production, infant milk intake, exclusive pumping, lactation, breastfeeding, human milk, breast pumping, breast expression, electric breast pump, expressed breast milk storage

## Abstract

**Highlights:**

**What are the main findings?**

- Milk production is higher in exclusively pumping women compared with fully breastfeeding and breastfeeding women who are occasionally pumping.
- The time commitment associated with exclusive pumping represents a serious burden.

**What are the implications of the main findings?**

- This study confirms it is possible to sustain lactation with exclusive pumping, providing sufficient expressed breast milk volumes for the infant.
- Exclusively pumping women will benefit from improved professional support and easily accessible evidence-based pumping and feeding guidelines.

**Abstract:**

**Background/Objectives:** Women who exclusively pump (EP) expend more time and effort on pumping and feeding infants their expressed breast milk (EBM) than those who directly breastfeed. However, little is known about their 24 h milk production (MP) or pumping and feeding dynamics. **Methods:** An online survey of 195 EP women explored the experiences, characteristics, MP and pumping and feeding dynamics of EP women. Quantitative data were compared by prematurity/neonatal unit admission, milk supply and parity, and self-reported MP was compared with MP in a predominantly breastfeeding reference group. **Results:** The 24 h pumping frequency was 6.0 [5.0, 8.0] with a pumping session duration of 25 [20, 30] min and a total pumping time of 2.3 [1.8, 3.0] h/24 h. The self-reported MP was 900 [600, 1100] mL/24 h, which was higher than that of the reference group (*p* = 0.012). Infant cues (58%), as well as advice from the child health nurse (39%), lactation consultant (34%) and pediatrician (34%) informed EBM feed volumes. Fifty-six percent of the infants were fed on demand, with 37% using a feeding schedule. Infants were fed 7.0 [6.0, 8.0] times/24 h; the average individual EBM feed and total 24 h volume were 120 [100, 150] and 750 [600, 900] mL, respectively. **Conclusions:** This study confirms it is possible to sustain lactation with EP and provide sufficient EBM volumes for the infant. However, the time commitment of EP and infant feeding represents a serious burden. These families would benefit from enhanced professional support and accessible evidence-based pumping and feeding guidelines.

## 1. Introduction

Breast milk expression is a method of removing milk from the breast by manual or electric breast pumps that apply a vacuum (pumping), or by hand expression with the application of positive pressure to the milk ducts [1]. Pumping is increasingly common with improved technology, breastfeeding difficulties, women returning to paid employment, and parents choosing to share infant feeding responsibilities or wanting to know infant milk intake [1,2,3,4,5,6]. Exclusive pumping (EP) is a method of providing expressed breast milk (EBM) for an infant instead of feeding directly at the breast, feeding it to the infant via several methods including syringes, tubes, cups and bottles [1,7,8,9,10]. The World Health Organization (WHO) definition of exclusive breastfeeding is that an infant receives only breast milk, including directly from the breast, expressed or donated milk, with no supplementation with solids or other liquids, with the exception of oral vitamins and/or medication [11].

Women who EP and their infants continue to receive multiple health benefits associated with breastfeeding, including reduced risk of maternal and infant cancers and obesity [12,13,14,15,16,17]. EBM feeding, similar to direct breastfeeding, is associated with better cognitive outcomes than commercial milk formula feeding [18,19]. Notwithstanding the benefits of breast milk, EBM, as well as commercial milk formula, is typically delivered via a bottle, and bottle-feeding is linked to reduced self-regulation of milk intake [20], accelerated weight gain [21,22,23], and greater risk of otitis media [24] and oral health complications irrespective of the type of milk fed [25,26].

Predominantly pumping and EP women have a high prevalence of maternal and infant health conditions and pregnancy complications, such as preterm and multiple births, which typically require NICU admission and are associated with direct breastfeeding and lactation challenges [1,6,27,28]. Despite the multiple challenges, including infant latching difficulties and breast refusal, which make EP women a high-risk population for early cessation of direct breastfeeding, these women demonstrate resiliency, seeking professional support when direct breastfeeding challenges arise and turning to EP when the issues cannot be resolved [6].

It is reported that many EP women do not receive adequate practical advice on pumping, or emotional support for the physical and mental workload involved in this time-consuming feeding method [5,29,30]. Despite this, a recent study of predominantly and EP women during established lactation in a high-income setting confirmed that the majority can maintain normal milk production (MP) [27] (denoted as ≥600 g/24 h [31]) with the use of efficient electric pumps (though not all women have access to them). Additionally, EP women have reported an intention of continuing EP for 12 months; of those who ceased EP around 6 months postpartum, 25% switched to direct breastfeeding [6].

Previous limited research has focused on the novelty of EP as a concept and EP women’s experiences and challenges [1,5,8,9,32]. The time-consuming nature of EP includes tasks such as pumping and feeding, as well as cleaning and maintenance of pumping equipment, EBM storage, and managing pumping around infant care, posing common EP challenges [1,5,6]. Professional support for EP is inconsistent; EP women report a lack of guidance on pumping frequency and duration, breast shield (flange) sizing and maintaining milk supply [6], forcing many EP women to research and access informal information from sources such as online support groups, where misinformation is frequent [5,9,32]. Finally, there is a lack of empirical data on EBM feeding, with no information on how much EBM and commercial milk formula (if supplementing) should be offered to growing infants of EP women, further exacerbated by the barriers to information seeking such as judgment or lack of vocabulary and terminology [9].

All these challenges are considerable when EP for a singleton infant. Those who EP for multiple infants, who are often premature, experience increased workload and parental concerns, particularly if they are struggling to pump enough milk for one infant. Whilst it is considered possible to breastfeed multiples due to the ‘supply and demand’ mechanism, where increased milk removal stimulates greater MP [33,34], data on the MP of women with multiple infants are rare [35,36]. Saint et al. showed increased volumes were produced by eight mothers of twins and one of triplets [37] compared with mothers of singletons, with the mother of triplets producing over 3 L/24 h of milk. However, one woman still had to supplement her infants to satisfy their energy requirements. Another study documented that six out of nine women with triplets self-reported they could breastfeed exclusively, including one woman who EP for 6 months, but three others combined direct breastfeeding, expressing and formula feeding [38]. Unfortunately, no infant growth data were reported.

There is an increasing need for research to inform clinical guidelines for health professionals to support EP families, who have different concerns and information needs than those directly breastfeeding or formula feeding [8,9]. EP women’s pumping and feeding characteristics and dynamics remain unexplored [1,9,39,40]. We used a mixed-methods approach and employed an online survey to collect data on EP women’s 24 h MP, pumping, feeding dynamics and logistics, as well as pumping equipment care and EBM storage. We recently reported the demographics, obstetric history, pregnancy complications, health and breastfeeding and expression challenges and experiences of the same cohort [6].

As access to information and experiences with clinical care and support may be different due to infant–mother separation, expectations of infant breastfeeding ability and access to lactation support [6,41], in this exploratory study we aimed to compare the quantitative data of EP women whose infants were born preterm (<37 weeks’ gestation) and had an NICU/neonatal unit admission with those of women with healthy term infants. Additionally, as breastfeeding outcomes differ by parity [42], we compared pumping dynamics of primiparous and multiparous EP women. Finally, we compared self-reported pumping and feeding data of EP women who reported 24 h MP <600 mL (low milk supply, LMS) [31,43] with those with normal milk supply (NMS).

## 2. Materials and Methods

### 2.1. Study Design and Participants

The study design, participants and survey questions have been described in detail previously [6]. Briefly, EP women were defined in this study as those only feeding their infant pumped/expressed breast milk, i.e., the infant does not receive breast milk directly from the breast. Women were still considered to EP if the infants also were fed formula. Participants completed an online anonymous 85-item survey using the secure web-based software platform designed to support data capture for research studies, Research Electronic Data Capture (REDCap, version 15.5.23), hosted at The University of Western Australia [44,45]. The experienced lactation and maternity care professionals and EP women were consulted in the design and development of this survey to ensure consumer engagement for validity and relevance. Quantitative items allowed for the collection of participant demographics, maternal and infant characteristics and pumping and feeding logistics; the typed responses had no word limit.

Participants were recruited for the survey through online social media posts on Facebook groups in Australia, the USA, the UK, New Zealand and France that targeted women with EP experience, including online peer-support groups for EP women and for parents of preterm/NICU infants. Inclusion criteria: English-speaking women ≥18 years old who currently were or had EP their breast milk to feed their infants who were <24 months old at the time of survey. Exclusion criteria: mothers of infants over 24 months of age at the time of survey. The reliability of maternal recall of birth and breastfeeding events within 24 months postpartum is generally considered to be high [46,47].

The survey was conducted in accordance with the Declaration of Helsinki and received ethical approval from The University of Western Australia Human Research Ethics Committee (2025/ET000304). Participants completed an online participant information and consent form, and digital consent was signed. Participants were informed of their right to withdraw from the survey at any time with no consequence and were assured of confidentiality and privacy. The survey took approximately 15 min to complete. This paper focuses on EP women’s 24 h MP, pumping and feeding dynamics and logistics, as well as pumping kit care and EBM storage.

### 2.2. 24 h Milk Production and Infant Milk Intake Determination

Participants self-reported their 24 h MP in response to the question “What is the total volume that you typically pump in 24 h? (Please state if in mL or oz)”. For the comparison of self-reported 24 h MP (mL) data of EP women with the general breastfeeding population, we used 24 h MP data measured by the test weighing method [31] by women who fully breastfed or pumped occasionally during established lactation (reference group; *n* = 59; 4.1 [3.0, 4.9] months postpartum) [27]. This study was also conducted in accordance with the Declaration of Helsinki and was approved by The University of Western Australia Human Research Ethics Committee (2019/RA/4/20/6134 and 2019/RA/4/20/6407), and informed written consent was obtained from all participants.

For the comparison, the reference group 24 h MP was converted from g to mL using the breast milk density of 1.03 g/mL [48]. Participants also reported the 24 h minimum and maximum amounts that they usually pumped, as well as their infant’s EBM and formula intake per feed and over 24 h. Additionally, the average milk volume pumped per session (mL) was calculated as self-reported total milk volume (mL) pumped in 24 h divided by the self-reported number of pumping sessions per 24 h.

### 2.3. Statistical Analysis

For this survey, a sample size of 90 was determined using the ‘F tests—Linear multiple regression: Fixed model: R^2^ increase option’ in G*Power 3.1 [49]. With one predictor, a small effect size (Cohen’s *f*^2^ below 0.1), and an alpha level of 0.05, a total sample size of 90 participants was determined to achieve a power of 0.80 [50]. The sample size was increased to 100 to account for missing data.

Continuous data were assessed for normal distribution using the Normality Test (Shapiro–Wilk) and Q-Q plot and described as mean ± standard deviation (SD) for normally distributed data or median [IQR] for skewed data. Categorical data were described by frequencies/counts and percentages.

**Figure 1:**
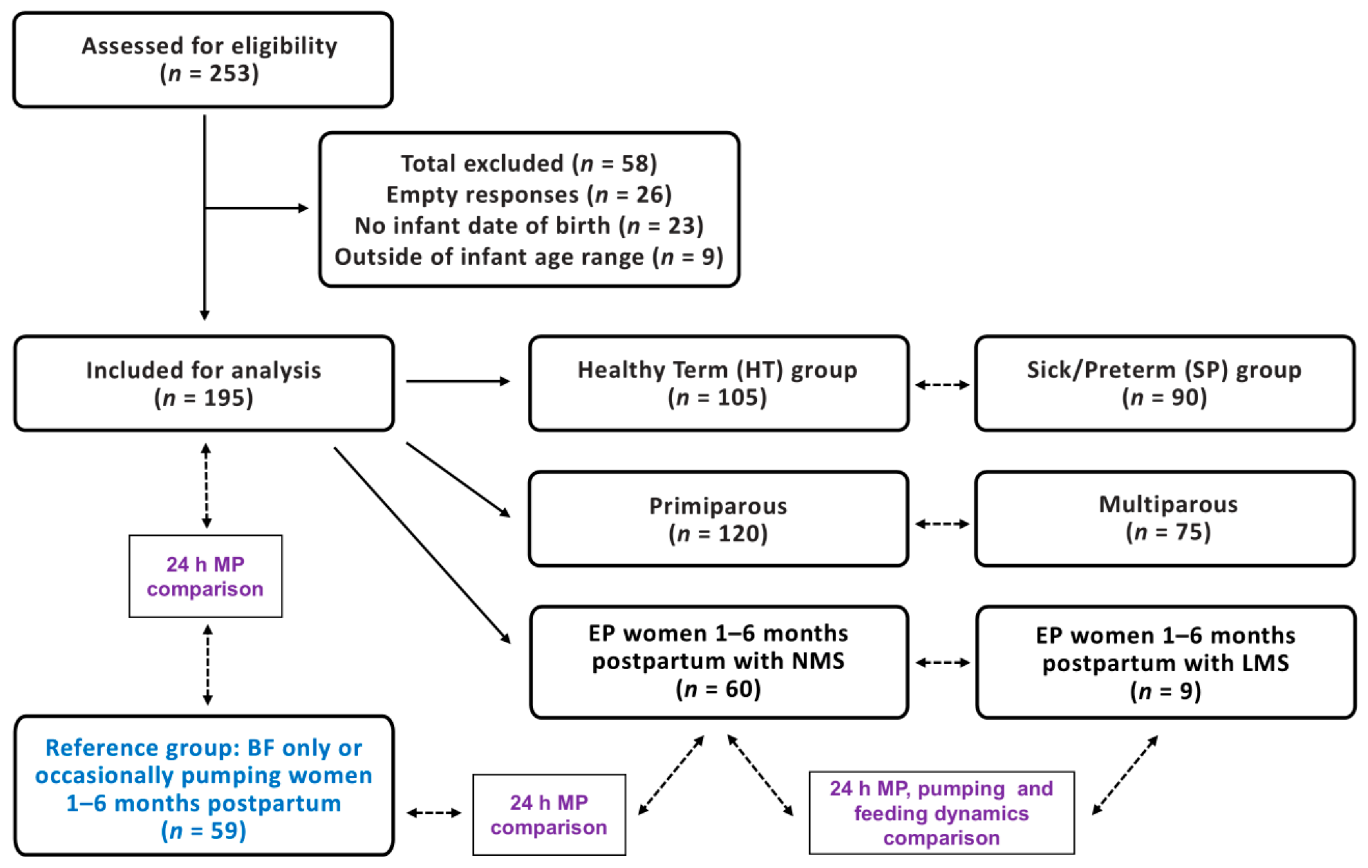
Study recruitment and between-groups comparison (dotted arrows) flow chart. BF, breastfeeding; HT, healthy term group—women with term infants who did not have a neonatal unit admission; LMS, low milk supply; NMS, normal milk supply; SP, sick/preterm group—women with infants who were born preterm (<37 weeks’ birth gestation) and/or had a neonatal unit admission. Blue font indicates Reference group consisting of women that breastfed and/or pumped occasionally [27]; purple font indicates comparisons conducted between the groups.

Students’ *t*-tests, the Chi-square test or the Fischer’s Exact test were used as appropriate to compare variables of women with term infants who did not have a neonatal unit admission (healthy term (HT) group) and those who’s infants were born preterm (<37 weeks birth gestation) and/or had a neonatal unit admission (sick/preterm (SP) group) (Figure 1). Additionally, we compared primiparous and multiparous EP women; only significant differences are reported.

Furthermore, we compared self-reported pumping and feeding data of EP women who reported 24 h MP <600 mL (LMS) with those with NMS. To avoid recall bias, for this comparison we included only those EP participants who were in established lactation at the time of the survey (between 1 and 6 months postpartum, when lactation and infant milk intake are stable) [31]. As the number of EP women with LMS was low (*n* = 9), we advise caution when interpreting these results.

An unpaired *t*-test was used to compare EP women’s self-reported 24 h MP (of the Total cohort and of those between 1 and 6 months postpartum) with the data of breastfeeding and/or occasionally pumping women reported in the Western Australian population (reference group) [27].

We also presented self-reported 24 h MP and infant milk intake data from a small group of EP women with twins (*n* = 14) as these data are scarce in the literature [36].

Missing data were addressed using available case analysis (pairwise deletion) to have higher efficiency, to preserve the sample sizes and to avoid bias and loss of precision [51]. Adjustments for multiplicity were not conducted in this exploratory study, and caution should be exercised when interpreting *p*-values; results were considered statistically significant when *p* < 0.05. All analyses were performed using R statistical software (version 4.4.2; R Foundation for Statistical Computing, Vienna, Austria).

## 3. Results

### 3.1. Participants’ Demographics and Expressing Status

Participants’ demographics and breastfeeding and EP characteristics (compared by parity and prematurity/NICU admission) have been published previously [6]. Briefly, of the 253 responses received, *n* = 195 met the study criteria; 120 were primiparous; and 105 were mothers of healthy term infants who were not admitted to a neonatal unit (HT group). The majority of women (58%) who completed the survey were EP at the time of the survey, and 55% (41/75) of multiparous women had EP during previous lactation; most women lived in Australia (92%), were married or in a de facto relationship (93%) and were tertiary educated (66%) (Table 1).

### 3.2. Pumping Dynamics and Milk Production in EP Women

#### 3.2.1. Whole Cohort

Forty-three percent (*n* = 84) of EP women reported experiencing perceived LMS during their lactation [6]. Most EP women double pumped (76%) and pumped at night (87%) (Table 2). The duration of pumping session was 25.0 [20.0, 30.0] min. The median self-reported 24 h MP (total milk volume pumped in 24 h) was 900 [600, 1100] mL/24 h (Table 2).

#### 3.2.2. Primiparous vs. Multiparous Women

Primiparous women pumped more frequently (6.0 [5.0, 8.0] vs. 6.0 [4.5, 7.0] sessions/24 h, *p* = 0.029) and pumped smaller EBM volumes per pumping session (135 [88, 200] vs. 167 [120, 224] mL, *p* = 0.034) than multiparous women.

#### 3.2.3. Women with Healthy Term vs. Sick/Preterm Infants

The SP group were more likely to single pump than the HT group (*p* = 0.009) (Table 2). They pumped more frequently (*p* = 0.001), had shorter session durations (*p* = 0.023) and pumped smaller volumes (*p* = 0.024) than the HT group. The median self-reported 24 h MP was 910 [650, 1100] mL/24 h in HT and 800 [550, 1140] mL/24 h in the SP group (Table 2).

#### 3.2.4. Women with Normal vs. Low Milk Supply

Of those who reported average 24 h milk volume pumped and were currently EP and 1–6 months postpartum at the time of survey (*n* = 69), 13% (9/69) had LMS (Table 2). Half of the LMS group and 40% of the NMS group reported LMS as a lactation challenge they have/had experienced, whereas 50% and 37% of the LMS and NMS group, respectively, selected it as a challenge of EP. The LMS group pumped for less time/24 h (*p* = 0.045) than the NMS group. The median self-reported 24 h MP was 900 [700, 1084] mL/24 h in the NMS group and 350 [325, 500] mL/24 h in the LMS group (Table 2).

#### 3.2.5. 24 h Milk Production Comparison

**Figure 2:**
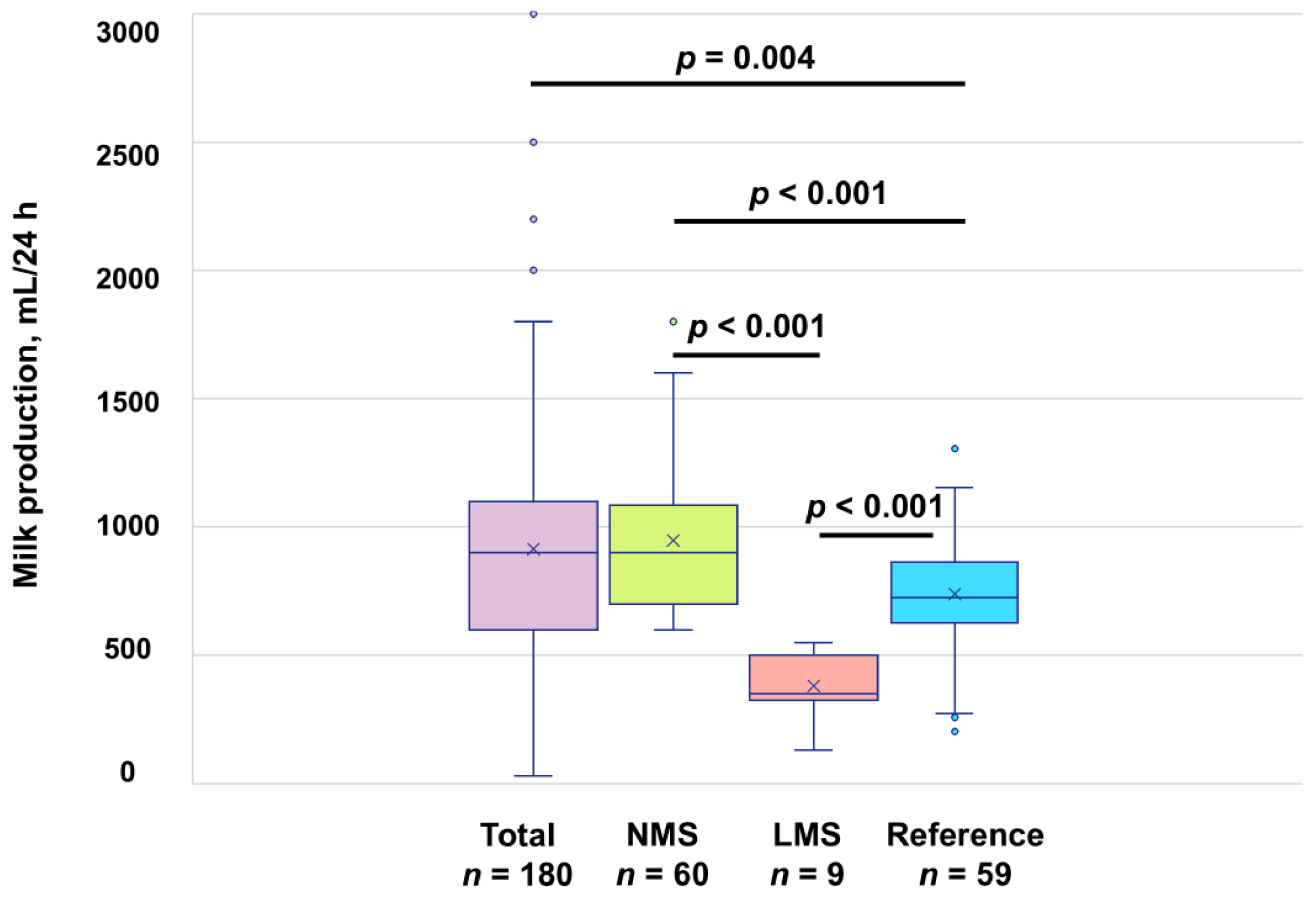
Comparison of self-reported 24 h milk production of exclusively pumping women (Total and with low (LMS) and normal milk supply (NMS) during established lactation) with milk production of the reference group of women who fully breastfed or pumped occasionally (Reference group [27]).

When compared to previously published 24 h MP data of women who fully breastfeed or pump occasionally (reference group: 738 ± 214 mL/24 h, *n* = 59 [27]), the 24 h MP of EP women in this cohort was higher (Total: *p* = 0.004, NMS: *p* < 0.001) (Figure 2). The 24 H MP in the LMS group was lower than in the NMS or the reference group (*p* < 0.001 for both).

#### 3.2.6. Women with Twins

**Figure 3:**
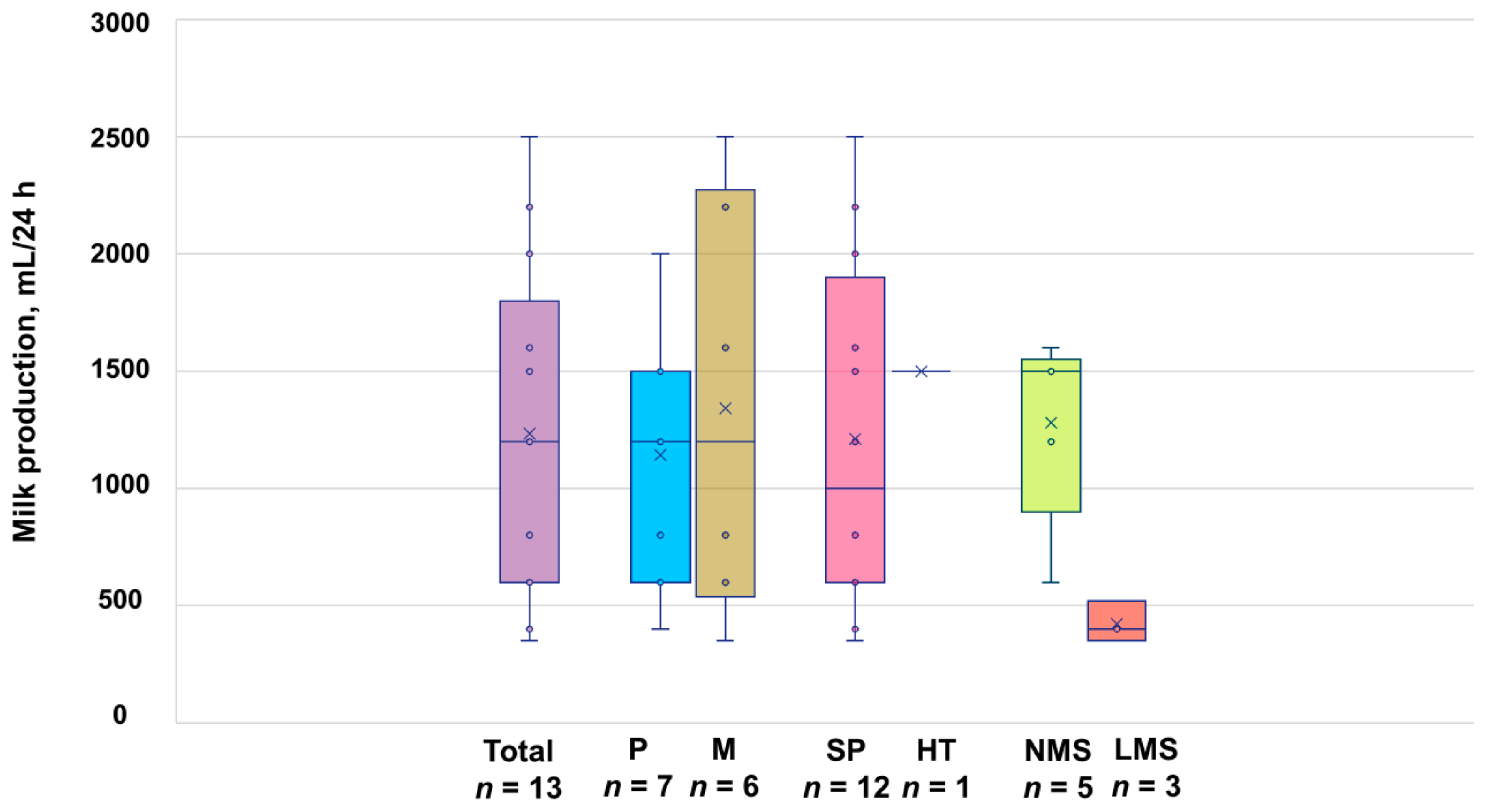
Self-reported 24 h milk production of women exclusively pumping for twins. The circles represent individual data. HT, healthy term group (women with term infants who did not have a neonatal unit admission); LMS, low milk supply; M, multiparous; NMS, normal milk supply; P, primiparous; SP, sick/preterm group (women with infants who were born preterm (<37 weeks birth gestation) and/or had a neonatal unit admission).

In EP women with multiple infants (twins) who self-reported 24 h MP at 6.3 [4.6, 13.6] months postpartum, the median 24 h MP was 1200 [600, 1800] mL/24 h in the Total group (*n* = 13); 1200 [600, 1500] mL/24 h in the primiparous group (*n* = 7); 1200 [538, 2275] mL/24 h in the multiparous group (*n* = 6); 1500 mL/24 h in the HT group (*n* = 1); 1000 [600, 1900] mL/24 h in the SP group (*n* = 12); and 1500 [900, 1550] mL/24 h in the NMS group (*n* = 5) (Figure 3).

### 3.3. Infant Feeding Information and Practices

#### 3.3.1. Whole Cohort

How much milk women fed their infant was derived from the infant’s cues (58%) and advice from the child health nurse (39%), lactation consultant and pediatrician (both 34%) (Table 3). Fifty-seven percent were fed on demand, and 37% used a schedule. Infants were fed 7.0 [6.0, 8.0]/24 h; 74% of infants were fed at night. Most shared the responsibility of feeding with their partner (63%). The average volumes of EBM consumed by infants per feed and per 24 h were 120 [100, 150] mL and 750 [600, 900] mL, respectively (Table 4). In the infants supplemented with formula (*n* = 56), the average volumes of formula per feed and per 24 h were 120 [100, 180] and 230 [150, 415] mL, respectively (Table 4). The total infant milk intake, including intake of EBM and formula, was 810 [700, 1000] mL/24 h (Table 4). Infants who started on formula and solid foods were introduced to them at the average age of 0.5 [0.03, 2.0] and 6.0 [5.0, 6.0] months of age, respectively (Table 4). Fourteen percent of women in the Total group introduced solids at 4 months.

#### 3.3.2. Primiparous vs. Multiparous Women

Multiparous EP women were less likely to seek infant feeding information from the child health nurse than primiparous women (19/65 (29%) vs. 49/111 (44%), *p* = 0.04988). Primiparous women fed more frequently than multiparous women (7.0 [6.0, 8.0] vs. 6.5 [5.0, 8.0] times per 24 h, respectively, *p* = 0.032) but not during the night (*p* = 0.99).

#### 3.3.3. Women with Healthy Term vs. Sick/Preterm Infants

The SP group were more likely to seek infant feeding information from the pediatrician (*p* < 0.001) and less likely to seek it from their general practitioner (*p* = 0.023), child health nurse (*p* = 0.011), websites (*p* = 0.015) and infant cues (*p* < 0.001) compared to the HT group (Table 3). The SP group infants were more likely to be fed on schedule (*p* < 0.001) and at nighttime (*p* = 0.016) than those in the HT group (Table 3). The SP group fed less EBM/feed (*p* < 0.001) and per 24 h (*p* = 0.001) and less formula/feed (*p* = 0.005) than the HT group (Table 4). The SP group infants had lower total 24 h milk intake than the HT group infants (*p* = 0.001) (Table 4). Further, the HT group infants were more likely to be introduced to solid foods before 6 months of age (*p* = 0.005) (Table 4), with 5% in the SP group and 20% in the HT group receiving solids at 4 months.

#### 3.3.4. Women with Normal vs. Low Milk Supply

The average volumes of EBM fed by the NMS group per feed and per 24 h were 120 [100, 150] mL and 750 [650, 820] mL, respectively (Table 4).

#### 3.3.5. Twins

Twin EBM intake was 725 [460, 850] mL/24 h (*n* = 9), commercial milk formula intake was 400 [175, 415] mL/24 h (*n* = 4), and total milk intake was 900 [820, 1125] mL/24 h (*n* = 7).

### 3.4. Pumping Equipment Details

The majority of EP women (82%) were using/had used more than one pump (1.4 ± 1.0, Min–Max: 1–5) (Table 5). The most-used pump types were a hospital-grade electric breast pump (47%) and wearable (27%) and personal electric breast pumps (22%). Women used predominantly medium (59%) vacuum settings. The median breast pump shield size used was 21 [19, 24] mm, and 68% of women reported that they used a pumping bra. The SP group were more likely to use a hospital-grade electric breast pump (*p* = 0.034) and to hand express (*p* = 0.021) than the HT group (Table 5).

### 3.5. Breast Pump Selection Details

Around half of EP women (53%) purchased their breast pump after birth (Table 6). However, more multiparous women purchased their breast pump during pregnancy than primiparous women (27/72 (38%) vs. 21/116 (18%), *p* = 0.004). Multiparous women also were less likely to receive the pump as a gift (2/72 (3%) vs. 8/116 (7%), *p* < 0.001) or to borrow one from a friend (4/72 (6%) vs. 14/116 (12%), *p* = 0.001). The pumps were chosen predominantly based on online reviews (42%), friends and family advice (36%) and social media (36%). The SP group were less likely to choose a breast pump based on friends/family (*p* = 0.005) or social media (*p* = 0.018) advice and more likely to choose the breast pump they had used in the NICU (*p* = 0.008). The ability of the breast pump to remove satisfactory (‘good’) volumes of milk (86%), mobility (63%) and comfort (62%) were the most important aspects of the breast pump for EP women. Additionally, mobility was a less important aspect of the breast pump for the SP group (Table 6). The most frequently stated improvement was pump price point (14%), followed by more flange size options (11%) and noise acceptability (7%) (Table A1).

### 3.6. Pumping Kit Storage and Cleaning Details

Over half of EP women stored their pumping kit in the refrigerator (58%) and washed the equipment with soap and water (85%), either after each use (50%) or once per day (45%) (Table 7). The SP group were more likely to store their pumping kit on the bench at room temperature (*p* = 0.010) and less likely to store it in the refrigerator (*p* < 0.001) compared to the HT group. They also were more likely to use a microwave (*p* = 0.026) and less likely to use a dishwasher for cleaning their pumping kit (*p* = 0.040). Additionally, the SP group were more likely to clean the pumping kit after each use (*p* < 0.001) and less likely to clean it once a day (*p* < 0.001). Two-thirds of EP women were responsible for the equipment cleaning and a third shared this responsibility with their partners (Table 7).

### 3.7. Expressed Breast Milk Storage Details

The average daily surplus of EBM (calculated as a difference between average EBM pumped in 24 h and average infant EBM intake/24 h) was 100 [0, 350] mL (Table 8). At the time of survey, the majority of EP women (77%) had some EBM stored either in a chest freezer (53%) or a fridge freezer (44%) (Table 8). The SP group were less likely to use a fridge freezer than the HT group (*p* = 0.026).

**Figure 4:**
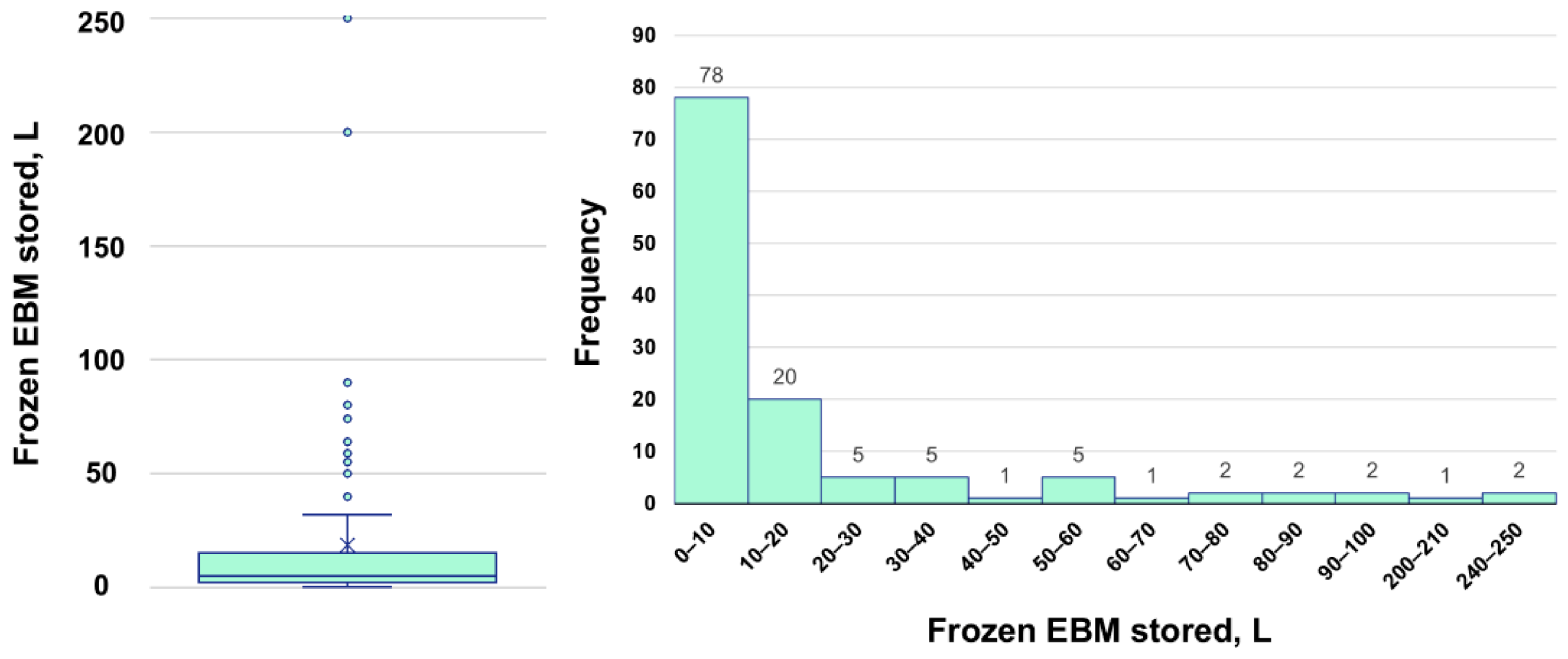
The currently stored amount of frozen EBM (*n* = 124); shown as a boxplot and frequency of distribution. EBM, expressed breast milk.

The plans for stored EBM were to use it when EP has ceased (60%), to supplement fresh EBM (23%) or for donation (16%). The currently stored amount of frozen EBM was 5.0 [2.0, 15.0] L (Figure 4). The EBM was frozen for a maximum of 6.0 [3.0, 10.0] months. The EP women were planning to cease pumping and feeding EBM when their infants reached 12 [7, 12] and 12 [8, 16] months of age, respectively (Table 8), the latter being significantly longer (0.0 [0.0, 3.0] months, *p* = 0.002).

### 3.8. Returning to Work and EP for Future Children

Most mothers were planning to return to paid work at 6–12 months (41%) or after 1 year post-birth (36%) (Table 9), with more primiparous EP women indicating that they would return to paid work after 1 year postpartum than multiparous women (45/109 (41%) vs. 16/62 (26%), *p* = 0.042). The SP group were less likely to return to work within 3–6 months of birth (*p* = 0.040) and more likely to return to work after 1 year (*p* = 0.016) than the HT group. Most women stated that returning to work did not influence EP (56%) or influenced it slightly (15%). Over two-thirds of women (69%) stated that they would consider EP for future children if direct breastfeeding was not possible (Table 9).

## 4. Discussion

This study confirms it is possible to sustain lactation with EP and provide sufficient EBM volumes for the infant. EP women in this study reported higher 24 h MP volumes than women who fully breastfed or pumped occasionally. Similarly, infant 24 h EBM intake was more than 750 mL. However, the time associated with EP and infant feeding represents a serious burden. Multiple concerns regarding milk removal, storage and feeding have been identified and remain to be addressed in formal guidelines and support for both women whose infants were born preterm and/or had a neonatal unit admission and women with healthy term infants.

### 4.1. Breastfeeding Duration and Milk Production in Exclusively Pumping Women

We have previously reported that EP women in this predominantly Australian cohort had strong intentions to breastfeed, with most intending to exclusively direct breastfeed or combine direct breastfeeding with pumping and EBM feeding, for a median of 12 months [6]. Those who completed the survey after ceasing EP stopped EP when their infants were a median of 6.0 months old, and those still EP were aiming at continuing to a median of 12.0 months. EP women self-reported high 24 h MP volumes of 900 mL and above in the Total, HT and NMS groups, challenging the long-held dogma that it is not possible to sustain lactation with EP and provide sufficient EBM volumes for the infant [52].

The previously reported shorter lactation duration and increased use of formula by EP women compared with those who directly breastfeed (even if they occasionally pump) have been partially explained in the past by the lower efficacy of milk removal with breast pumps compared to breastfeeding, resulting in decreased MP [4,8,39,40,52]. In contrast, EP women from this cohort had longer lactation durations, similar to a large EP USA study that reported a mean duration of 9 months, comparing favorably with those directly breastfeeding [9]. Importantly, they reported MP volumes higher than breastfeeding only or occasionally pumping women and above the average global milk intake of exclusively breastfed 3–6-month-old infants (758 mL/24 h) [53]. Our participants were from high-income settings, and most of them used either a hospital-grade electric breast pump or a wearable or personal electric breast pump (Table 5); thus, these pumps’ better efficacy would likely be conducive to establishing and maintaining a full MP.

These findings are supported by the previous report of EP women from Western Australia having a 24 h average percentage of available milk removed (PAMR) with home breast pumps similar to that of breastfeeding only and higher than in occasionally pumping women (breastfeeding only: 75.8 ± 13.2%; EP: 83.0 ± 8.4%; occasionally pumping: 70.0 ± 10.9%; *p* = 0.012) [54]. Further, PAMR with a personal electric breast pump has been shown to be higher than that of infants breastfeeding to appetite (personal electric breast pump PAMR: 73.6 ± 32.1%; breastfeeding infants PAMR: 67.3 ± 7.8%; *p* < 0.001) [55]. PAMR is considered a gold standard measure of the effectiveness of milk removal; it provides a more accurate measure of breast drainage compared to absolute volume removed, as it takes into account the volume of milk in the breast prior to expression [4]. The difference in PAMR is most likely due to infants feeding to appetite and the fact that infants may at some point in the day remove more milk than a pump if the breast is fuller [56,57]. These studies together with our findings in this cohort suggest that when direct breastfeeding is not possible, pumping with an effective breast pump may sustain MP for a considerable period of time.

Despite the overall high 24 h MP volumes, 43% of women reported LMS and 42% perceived it as a challenge, with 11% acknowledging it contributing to the decision to EP [6] and 32% needing to supplement formula. Rates of perceived LMS in this cohort are similar to those of predominantly direct breastfeeding in the Russian Federation (39%) [58], Western Australia (44%) [59] and globally (35%) [60]. Clinically, it is still assumed that only 5% of women have LMS [61] despite recent evidence that this number is around 20–30% [62,63]. Further, the current definition of LMS lacks consensus and methodological rigor, relying on somewhat arbitrary thresholds, such as <600 mL/24 h [31,43], which is substantially lower than the average breastfed infant’s milk intake that is closer to 750 mL/24 h [31,53]. A more nuanced, data-driven classification of milk supply has been recently proposed, which identified new thresholds of 708 g/24 h for MP and 694 g/24 h for infant milk intake [64]. The proposed threshold of 708 g/24 h for MP may absorb some of the above-reported LMS as true. In those who were currently EP at 1–6 months postpartum, using the new evidence-based threshold of 708 g/24 h would increase the rate of LMS from 13% to 29%. Of some concern is also that LMS was underreported in the LMS group and overreported in the NMS group, with around half of women in each group selecting LMS as a lactation or EP challenge they had experienced. It was reassuring that there was no difference in reported 24 h MP between the SP and the HT groups. The absence of information regarding normal MP may have a negative impact on EP families, who may mistakenly determine pumped breast milk volumes as adequate. Further, they may subsequently freeze a substantial volume (breast milk ‘stash’) rather than offer it to the infant [8,40,65,66] which may partially explain the higher likelihood of stopping EP and breast milk feeding [40,65,66].

In a small group of women who EP for twins, 24 h MP was higher (median 1200 mL). However, given most infants require more than 600 mL/day, it is not surprising that 50% of these EP women had to supplement with formula to ensure adequate infant growth. Whilst it is commonly considered physiologically possible for women to produce an adequate milk supply for multiple infants due to the ‘supply and demand’ mechanism, with increased milk removal stimulating increased MP [33,34], this is often not the case. A recent Australian study reported that only 53% of women fully breastfed their multiple infants at some point during their lactation and that 49% had LMS concerns [36]. The psychological distress of looking after multiple infants may impair the release of oxytocin, leading to impairment of milk ejection and decreased MP due to incomplete emptying of the breast during each feed [67]. Further, multiple factors, such as breast hypoplasia [68,69], maternal age and obesity [70], as well as diabetes, polyendocrine metabolic ovarian syndrome and hypothyroidism [71,72], can impact breast development and reduce MP [64,73].

### 4.2. Infant Feeding in Exclusively Pumping Women

Quantifying MP [40,52] and wanting to monitor their infant’s milk intake [6,9,40] are some of the frequently reported reasons for EP. Approximately one third of participants in both a USA study [9] and in our cohort [6] wanted to monitor their child’s milk intake; this is distinct from the need to monitor intake for a slow infant weight gain (6%) or specific infant health conditions (22%) [6].

How much and how often milk should be pumped and provided to the infant are most frequently asked by EP women online [66]. EP women’s infants in this cohort were fed a median of 7.0 times per 24 h, with most feeding at night. This is similar to that of breastfeeding infants during established lactation, with a breastfeeding frequency of eight feeds at 1 month and seven feeds at 6 months of age [74]. The median volume of EBM fed per 24 h was 750 mL/24 h, which is similar to previously reported milk intake of exclusively breastfed infants [31,53]; however, total milk intake was ≥800 mL/24 h in most groups, indicating that some infants were not satisfied with EBM alone. Indeed, this is close to the mean average of 780 mL reported across four different countries [75]. USA women have articulated that their online research is often unproductive when it comes to practical and/or logistical aspects of EP, particularly on how much EBM and formula (if any) to feed their infant, despite many resources stating the needs of breastfed or formula-fed infants [9]. Further, despite no difference in reported 24 h MP, the SP group reported feeding less EBM and formula per feed, resulting in less EBM intake and total milk intake per 24 h, warranting further investigations. Our findings may contribute to accessible evidence-based pumping and feeding guidelines for EP families [66].

Although the majority of infants were fed on demand, a quarter were still feeding to a schedule. In this group of EP women, however, only 32% were supplementing with commercial milk formula, compared to 62% of Australian infants reported to receive formula or non-human milk at the age of 6 months [76]. Most parents relied on infant cues as well as health professional advice when determining what milk volumes to feed. However, women who experienced preterm birth and/or neonatal unit admission (SP group) were less likely to use infant cues and more likely to seek health professionals’ advice and use a feeding schedule in comparison to women with healthy infants. Their greater reliance on professional advice is potentially due to the differences in infant health and nutritional needs as well as parental experience and support associated with infant–mother separation [41]. This underpins the need for evidence-based information to be easily accessible by health care professionals.

In our study, infants who required formula supplementation received this during early lactation, when LMS concerns are at their highest. In contrast, the introduction of solid foods occurred around 6 months of age, in line with the WHO’s recommendations [77]. This is surprising, given the documented trend of Australian women to introduce solids at around 5 months, with a quarter of infants receiving solid foods between 4 and 5 months [78]. Interestingly, the SP group were more likely to introduce solid foods around 2 weeks later than the HT group, suggesting they are more likely to follow the guidelines and rely on professional advice.

Sharing infant feeding is a common reason for EP [6,9], and in this cohort the majority shared the responsibility with their partner, although this proportion reduced with increased parity. It has been shown that coparenting relationships may prevent maternal depression during the early postpartum period [79], and sharing infant feeding can reduce the physical, mental and emotional burden of EP, potentially preventing/lessening postnatal depression [9,80]. However, when others feed the infant, there is less ‘reward’ associated with infant feeding, such as positive interactions with the infant. This alters the traditional dyad interaction, which may actually contribute to maternal stress and low self-esteem [52,81]. The altered dyad interaction may result in less adaptive infant responses to stress and cortisol secretion, potentially leading to poorer outcomes including behavioral problems [82,83,84]. It has been suggested that using efficient hands-free pumps that can be worn under clothing (‘wearable pumps’) may offset mother–infant separation during pumping, leaving women free to interact with the infant or perform other tasks [1].

### 4.3. Pumping Dynamics and Logistics in Exclusively Pumping Women

The women in this study pumped for 20–25 min irrespective of the group. It is a common belief that EP women need to pump for longer; however, a recent Western Australian study reported that predominantly pumping and EP women could stop pumping after 12 min based on measures of milk removal [27]. This is similar to occasionally pumping women, in which only small volumes were removed (0 – <10% of total milk removed) after 12 min in single breast expression, as the first, most productive milk ejections typically occur within 10 min of the onset of milk flow [85].

The common belief that breast pumps are less effective compared to breastfeeding [4,52] is partially explained by older data showing breastfeeding infants can remove up to 80% of consumed milk in 5 min [86], when an efficient pump can remove 80% of available milk in 8 min during (simultaneous) breast expression [57]. However, the consumed and available milk can be considerably different. Further, infants are unable to remove milk from both breasts simultaneously, and usually do not feed for 5 min. There is a high variability in breastfeeding behavior ranging from 36 min at 1 month to 29 min at 6 months of age, lasting between 12 and 67 min [74]. The breastfeeding frequency is also variable, ranging from 4 to 13/24 h among infants (8/24 h at 1 month and 7/24 h at 6 months of age) [74]. EP parents are usually advised to pump at the same frequency as their infant would feed at the breast, expecting to pump 8–12 times daily, at least during early lactation [8]. However, the median number of double pumping sessions in this cohort was six, indicating MP can be sustained with fewer pumping sessions, likely due to the pump emptying the breast consistently during pumping sessions compared to the infant that feeds to appetite.

In our study, EP women spent 2.3 h/24 h pumping. To increase efficiency, EP women could familiarize themselves with their milk flow pattern, which is similar throughout lactation and between the breasts [87], to shorten their pumping session durations by not pumping when milk is no longer flowing. Avoiding extended pumping sessions also could be beneficial for reducing nipple trauma [88,89]. Use of a high-quality double electric pump is reported to be of high importance to parents who are reliant upon the breast pump [40], yet almost a quarter of the women in our study single pumped or alternated single and double pumping. It has been shown that on average, 10% more of the available milk is removed during simultaneous breast expression compared with expressing one breast after the other [57]. Therefore, there is an opportunity to improve pumping efficiency with simultaneous pumping using a hospital-grade electric double pump [90]. Interestingly, whilst the SP group were more likely to single pump and expressed smaller volumes in a single pumping session than the HT group, they pumped more frequently with a shorter session duration than the HT group.

It has been established that the increased strength of vacuum increases pumping efficacy [91], yet only 25% of women used strong vacuums, with the majority using either medium or weak vacuums during pumping. Whilst 50% of women prefer a maximum comfort vacuum of approximately −200 mmHg [91], predominantly and EP women may choose ~15% weaker vacuums (−187 ± 56 mmHg) than breastfeeding-only and occasionally pumping women (−221 ± 34 mmHg) [27]. The weaker vacuums during frequent pumping may be a strategy to avoid nipple trauma but may also lower efficacy, extending active flow durations and thus needing more milk ejections to remove the milk. Nevertheless, total flow duration, time to stop pumping and PAMR were similar to the reference group [27], indicating that even using weaker vacuums, longer pumping sessions with EP are not necessarily needed. Based on this, shorter pumping sessions with stronger vacuums (maximum comfort vacuum) might improve efficacy in EP women, provided no nipple pain is experienced, as this may inhibit milk ejection and milk flow [88,89]. Further research may elucidate individualized optimal pumping patterns for EP women.

### 4.4. Pump Selection and Use

Whilst half of EP women purchased their breast pumps after birth, 25% procured a pump in pregnancy. Conversely, a study of new mothers in Hong Kong reported that 80% of new mothers acquired a pump while pregnant or during their hospital stay [92], with 14% choosing EP for their infants. Efficacy of the breast pump was rated as the most important, including removal of satisfactory milk volumes and speed. Interestingly, pump choice was based predominantly on online reviews, friends and family advice and social media, with most healthcare professionals, except for lactation consultants, having little impact. These results demonstrate a generational/cultural change from seeking breast pump recommendations from healthcare personnel [66] to the ubiquity of information available. This is further exacerbated by the virtual absence of information regarding efficacy from most pump manufacturers [9]. On the contrary, the SP group were less likely to choose a breast pump based on friends/family and social media advice, and more likely to choose the breast pump they had used in the NICU and use a hospital-grade electric breast pump than the HT group, though there was no difference in hospital-grade pump use as the primary equipment after discharge.

Mobility and comfort during pumping were also top priorities when choosing pumps. It has been anecdotally suggested that wearable breast pumps are the cause of increasing EP rates [8,52], yet less than a third of our participants primarily used a wearable pump, with the majority using a hospital-grade pump. Whilst wearable/hands-free pumps allow maternal interaction with children and facilitate other tasks while pumping, little information exists on their efficacy [55], fueling skepticism among healthcare professionals [52].

Cost of pumping supplies was a challenge for half of participants [6] and rated highest as the most needed ‘pump improvement’, followed by more flange size options. Interestingly, most participants used more than one pump, supporting previous reports of EP parents being proficient in combining equipment from different manufacturers to suit their needs [8].

### 4.5. Pumping Kit Care and EBM Storage

EP women experience considerable challenges associated with pumping, feeding, cleaning and sterilizing pumping equipment, as well as milk storage [1]. In this cohort, EBM storage along with thawing of frozen milk were reported among other EP challenges, including lack of knowledge in general [6]. We found that most EP women stored their pumping kit in the refrigerator. We have no information on whether the breast shield was removed prior to storage; however, pumping with a cold breast shield may cool the nipple and areola and may slow initial milk flow due to reduction in milk duct diameters and contraction of the smooth muscle in the nipple [93]. Further, pumping with breast shields warmed up to 39 °C before expression has shown higher milk removal efficiency, particularly if pumping for a short time (5 and 10 min), achieving 80% PAMR faster than with a room-temperature breast shield (25 °C) [93]. Thus, removing the breast shield prior to pumping kit storage or warming the shield to room temperature may potentially decrease pumping time.

Interestingly, women from the SP group were less likely to store their pumping kit in the refrigerator and more likely to clean the pumping kit after each use rather than once a day and use a microwave for cleaning their pumping kit compared to the HT group. This could be due to the experience of increased hygiene and infection control measures required in the NICU/neonatal unit setting [94]. However, using soap and water was the preferred method for pumping kit cleaning by all groups.

There was a wide range of surplus EBM in this cohort (median 100 mL/24 h), with three quarters of women able to store their milk, either for later use when they ceased pumping, to supplement their fresh EBM if needed, or for donation. The median frozen EBM amount was 5.0 L, with 15 women storing EBM amounts above 50 L, one woman storing 200 L and two women storing 250 L. The most common reason for having an EBM ‘stash’ is that it gives parents reassurance that milk is available for temporary problems with milk supply, separation from infants, or illnesses [1,60,95]. As such, women who EP plan for and use their EBM ‘stash’ to meet their breastfeeding goals [8,96]. Interestingly, the breastfeeding duration is underestimated in EP women by at least a month, as they usually report their breastfeeding duration as the length of lactation rather than the length of time their infants receive EBM [9,97]. Indeed, our participants had a discrepancy between the intended length of EP and intended length of EBM feeding.

EP parents have multiple concerns regarding storage of EBM, including the length of time EBM can be safely stored for at room temperature, in the refrigerator and in the freezer and what effects freezing may have on their milk and infant outcomes [9]. Online information is conflicting and confusing regarding guidelines for EBM storage [98], and guidelines differ between countries. The Centers for Disease Control and Prevention (CDC, USA) recommends storing EBM in the freezer (−18 °C) for up to 6 months, whilst up to 12 months is acceptable in the deep (chest) freezer (−20 °C) [99]. Australian guidelines recommend a maximum of 2 weeks in a freezer compartment inside the refrigerator (−15 °C), 3 months in the freezer section of a refrigerator with a separate door (−18 °C) and 6 to 12 months in a deep freezer (−20 °C) [100].

There is sparse research into the effects of exclusive breast milk feeding and feeding of stored EBM on infant growth and development. Our participants kept EBM for a median of 6 months; however, some participants stored their milk up to 24 months, double that of the recommended time [101]. Whilst EBM can be safely stored for extended periods [102,103], more studies are needed to confirm its compositional integrity [98], with reports of reductions in milk fat and protein concentrations and activity [101,102,104].

### 4.6. Returning to Work and Plans to EP in the Future

EP women are more likely to be employed or plan to return to work soon after birth (1.5–4.5 months postpartum) in countries such as Hong Kong and the USA [29,92,105]. This is usually driven by the lack of or minimal paid maternity leave [106]. Interestingly, longer maternity leave in Australia (6 months) compared with the USA has not necessarily reduced EP prevalence [52], with rates of EP being similar in both countries (7% in Australia [27] and 6–14% in the USA [39,96,107]). This suggests that EP is likely more driven by maternal health, pregnancy complications and/or some breastfeeding challenges like latching issues and low MP [6].

Most EP women in this study were planning to return to paid work at 6–12 months or more than 1 year after birth, with primiparous women and the SP group more likely to return to work later. As on-site childcare is not an option at most workplaces, the majority of breastfeeding Australian women (71%) express breast milk at work [108]. In EP women, pumping skills and routines are already well established, making transition to part- or full-time employment easier. Effective wearable breast pumps may further reduce the challenges associated with pumping in the workplace [109], such as privacy and the need for lactation breaks.

Two-thirds of women who intended to have more children said that they would consider EP if their next infant could not be directly breastfed. Further, more than half of the multiparous women reported that they had already EP for their previous child. This commitment highlights that EP women value the benefits of human milk and lactation for both themselves and their children [17,30,110] and emphasizes the need for evidence-based guidelines on EP and infant feeding for this population.

### 4.7. Study Strengths and Limitations

The main strengths of this study are the large sample size and a series of questions on pumping and feeding dynamics and logistics that so far have been missing from research focusing on EP women, capturing invaluable data that can inform evidence-based pumping and feeding guidelines. This study provides a rare view of post-discharge maternal lactation experience with EP in women whose infants were preterm and/or admitted to the neonatal unit. However, our study also had some limitations [6]; it was an online survey, and due to self-selection, our participants could be biased [111]. Most of our participants had tertiary education, and, as such, results from this study may not apply to EP women with lower education levels and/or from low-income settings. Higher education is positively associated with higher socio-economic status [112], improved breastfeeding outcomes [113] and greater access to effective electric breast pumps [114].

Further, the majority of our participants were Australian, despite the survey being available globally; therefore, our findings are unlikely to be representative of the experiences of women from linguistically and culturally diverse or disadvantaged populations.

Furthermore, the primary outcome, 24 h MP, was obtained through participant self-report using the survey question “What is the total volume that you typically pump in 24 h?” rather than through direct measurement. However, the data measured in predominantly breastfeeding women using a validated reference method may still underestimate infant milk intake by 3–10% due to insensible water loss [115,116]. Exclusively pumping women constitute a unique population because they measure their 24 h MP on a daily basis and know their usual expressed volumes, information that is vital for feeding their infants; thus, we expect the bias to be minimal.

Finally, whilst the reliability of maternal recall regarding birth and breastfeeding events within 24 months postpartum is generally considered high [46,47], the pumping and feeding data, including 24 h MP volumes, were self-reported by participants at various postpartum time points, and it was not always clear to which time point during their lactation they were referring when answering these questions. Nevertheless, we could identify a modest subsample of EP women between 1 and 6 months postpartum, and answers outside this time frame could only contribute to a reduction in some of the reported values in other subgroups rather than inflate them. Further research focusing on EP women during established lactation would benefit our knowledge of their MP and pumping and feeding dynamics.

## 5. Conclusions

This study indicates that it is possible to sustain lactation with EP and provide sufficient EBM volumes for the infant. The time commitment with EP and infant feeding represents a serious burden. These women will benefit from enhanced professional support and easily accessible evidence-based pumping and feeding guidelines.

## Figures and Tables

**Table 1:** Participants’ demographics, exclusive pumping status and infant age at cessation of exclusive pumping.

Characteristics	Total *n* = 195	HT Group *n* = 105	SP Group *n* = 90	*p*-Value ^2^
Country				0.26
Australia	180 (92.3) ^1^	93 (88.6)	87 (96.7)	
United States of America	8 (4.1)	6 (5.7)	2 (2.2)	
United Kingdom	3 (1.5)	2 (1.9)	1 (1.1)	
New Zealand	3 (1.5)	3 (2.9)	0 (0.0)	
France	1 (0.5)	1 (0.9)	0 (0.0)	
Education level				0.88
High school	22 (11.3)	13 (12.4)	9 (10.0)	
Certificate/diploma	45 (23.1)	24 (22.9)	21 (23.3)	
Bachelor’s degree or above	128 (65.6)	68 (64.8)	60 (66.7)	
Marital status				0.099
Never married or de facto	10 (5.1)	7 (6.7)	3 (3.3)	
Married or de facto	182 (93.3)	98 (93.3)	84 (93.3)	
Separated or divorced	3 (1.5)	0 (0.0)	3 (3.3)	
Infant age at survey (months)	7.1 [3.9, 14.0]	6.3 [3.4, 11.9]	8.1 [3.9, 16.6]	0.061
Primiparous	120 (61.5)	60 (57.1)	60 (66.7)	0.17
Birth gestation (weeks)	38.6 [35.3, 39.4]	39.1 [38.7, 40.1]	34.6 [30.1, 37.6]	**<0.001**
EP status				0.96
Currently EP	113 (58.0)	61 (58.1)	52 (57.8)	
Previously EP	82 (42.0)	44 (41.9)	38 (42.2)	
Infant age at EP cessation (months) ^3^	6.0 [4.0, 9.0]	6.0 [4.0, 9.0]	5.0 [3.0, 10.5]	0.67

^1^ Data are *n* (%) or median [Q1, Q3]. ^2^ *p*-value indicates significant difference between HT (healthy term group—women with term infants who did not have a neonatal unit admission) and SP (sick/preterm group—women with infants who were born preterm (<37 weeks birth gestation) and/or had a neonatal unit admission) groups using unpaired Student’s *t*-test, Chi-square or Fisher’s exact test where appropriate. ^3^ For those that ceased EP prior to survey participation, *n* = 81. EP, exclusive pumping.

**Table 2:** Pumping dynamics and milk production in exclusively pumping women.

Characteristics	Total *n* = 190	HT Group *n* = 104	SP Group *n* = 86	*p*-Value ^2^	NMS *n* = 60	LMS *n* = 9	*p*-Value ^3^
Do/did you single or double pump?				**0.023**			**1.00**
Single	17 (9.0) ^1^	4 (3.9)	13 (15.1)	**0.009**	6 (10.0)	0 (0.0)	
Double	145 (76.3)	85 (81.7)	60 (69.8)	0.061	47 (78.3)	8 (88.9)	
Alternate single and double pumping	28 (14.7)	15 (14.4)	13 (15.1)	1.00	7 (11.7)	1 (11.1)	
Pumping sessions per 24 h	*n* = 187	*n* = 102	*n* = 85		*n* = 59	*n* = 9	
	6.2 [5.0, 8.0]	6.0 [5.0, 7.0]	7.0 [5.3, 8.0]	**0.001**	6.0 [5.0, 7.0]	4.5 [3.3, 7.5]	0.11
Nighttime pumping	*n* = 190	*n* = 104	*n* = 86		*n* = 60	*n* = 9	
Yes	165 (86.8)	88 (84.6)	77 (89.5)	0.32	49 (81.7)	8 (88.9)	1.00
Pumping sessions per night ^4^	*n* = 158	*n* = 84	*n* = 74		*n* = 48	*n* = 7	
	2.0 [1.0, 2.0]	1.3 [1.0, 2.0]	2.0 [1.0, 2.0]	0.072	1.3 [1.0, 2.0]	1.0 [1.0, 2.0]	0.40
Average pumping session duration (min)	*n* = 189	*n* = 104	*n* = 85		*n* = 60	*n* = 9	
	25.0 [20.0, 30.0]	25.0 [20.0, 30.0]	20.0 [15.0, 30.0]	**0.023**	20.0 [20.0, 30.0]	20.0 [17.5, 25.0]	0.23
Total 24 h pumping duration (h) ^5^	*n* = 187	*n* = 102	*n* = 85		*n* = 59	*n* = 9	
	2.3 [1.8, 3.0]	2.4 [2.0, 3.0]	2.3 [1.7, 3.0]	0.38	2.1 [1.8, 2.8]	1.7 [1.0, 2.7]	**0.045**
Minimum milk volume pumped (mL)	90 [50, 148]	100 [60, 150]	80 [40, 110]	0.13	100 [80, 150]	50 [25, 90]	**0.010**
Maximum milk volume pumped (mL)	*n* = 185	*n* = 102	*n* = 83		*n* = 60	*n* = 9	
	250 [160, 350]	255 [200, 380]	210 [150, 340]	0.098	250 [200, 350]	150 [120, 190]	**0.001**
Total 24 h milk volume pumped (mL)	*n* = 180	*n* = 98	*n* = 82		*n* = 60	*n* = 9	
	900 [600, 1100]	910 [650, 1100]	800 [550, 1140]	0.84	900 [700, 1084]	350 [325, 500]	**<0.001**
Average volume pumped per session (mL) ^6^	*n* = 179	*n* = 97	*n* = 82		*n* = 59	*n* = 9	
	147 [100, 211]	150 [119, 228]	129 [81, 200]	**0.024**	150 [117, 220]	75 [56, 119]	**0.002**

^1^ Data are *n* (%) or median [Q1, Q3]. ^2^ *p*-value indicates significant difference between HT (healthy term group—women with term infants who did not have a neonatal unit admission) and SP (sick/preterm group—women with infants who were born preterm (<37 weeks birth gestation) and/or had a neonatal unit admission) groups, ^3^ *p*-value indicates significant difference between LMS and NMS groups using unpaired Student’s *t*-test or Fisher’s exact test where appropriate. Bold font indicates a significant difference. LMS, low milk supply (<600 mL/24 h) between 1.0 and 6.0 months postpartum; NMS, normal milk supply between 1.0 and 6.0 months postpartum. ^4^ Nighttime is from 10:00 p.m. to 04:00 a.m. ^5^ Calculated as self-reported number of pumping sessions per 24 h multiplied by self-reported average pumping session length (min). ^6^ Calculated as self-reported total milk volume (mL) pumped in 24 h divided by self-reported number of pumping sessions per 24 h.

**Table 3:** Exclusively pumping women’s infant feeding details.

Characteristics	Total *n* = 176	HT Group *n* = 98	SP Group *n* = 78	*p*-Value ^2^
Infant feeding information source ^3^				
Infant cues	102 (58.0) ^1^	69 (70.4)	33 (42.3)	**<0.001**
Child health nurse	68 (38.6)	46 (46.9)	22 (28.2)	**0.011**
Lactation consultant	59 (33.5)	34 (34.7)	25 (32.1)	0.85
Pediatrician	59 (33.5)	18 (18.4)	41 (52.6)	**<0.001**
Midwife/OBGYN nurse	35 (19.9)	22 (22.5)	13 (16.7)	0.34
Websites	30 (17.1)	23 (23.5)	7 (9.0)	**0.015**
Social media	18 (10.2)	13 (13.3)	5 (6.4)	0.21
Online forums	15 (8.5)	11 (11.2)	4 (5.1)	0.18
General practitioner	14 (8.0)	12 (12.2)	2 (2.6)	**0.023**
Obstetrician	6 (3.4)	3 (3.1)	3 (3.9)	1.00
Friends/family	5 (2.8)	5 (5.1)	0 (0.0)	0.067
Did not seek information	12 (6.8)	6 (6.1)	6 (7.7)	0.77
Infant feeding practices				**<0.001**
On demand	100 (56.8)	67 (68.4)	33 (42.3)	**<0.001**
Feeding schedule	65 (36.9)	23 (23.5)	42 (53.9)	**<0.001**
Other	11 (6.3)	8 (8.2)	3 (3.9)	0.35
Number of feeds in 24 h	*n* = 173	*n* = 98	*n* = 75	
	7.0 [6.0, 8.0]	7.0 [6.0, 8.0]	7.0 [6.0, 8.0]	0.19
Infant fed at night (yes) ^4^	*n* = 176	*n* = 98	*n* = 78	
	131 (74.4)	66 (67.4)	65 (83.3)	**0.016**
Night feeding frequency ^4^	*n* = 129	*n* = 66	*n* = 62	
	2.0 [1.0, 2.0]	1.8 [1.0, 2.0]	2.0 [1.0, 2.1]	0.097
Infant feeding responsibility				0.45
Shared with partner	110 (62.5)	61 (62.3)	49 (62.8)	
Mostly me	62 (35.2)	36 (36.7)	26 (33.3)	
Mostly partner	4 (2.3)	1 (1.0)	3 (3.9)	

^1^ Data are *n* (%) or median [Q1, Q3]. ^2^ *p*-value indicates significant difference between HT (healthy term group—women with term infants who did not have a neonatal unit admission) and SP (sick/preterm group—women with infants who were born preterm (<37 weeks birth gestation) and/or had a neonatal unit admission) groups using unpaired Student’s *t*-test, Chi-square or Fisher’s exact test where appropriate; bold font indicates a significant difference. ^3^ Sum of percentages > 100%, as participants could select more than one response. ^4^ Nighttime is from 10:00 p.m. to 04:00 a.m. OBGYN, obstetrics and gynecology.

**Table 4:** Expressed breast milk and other food fed to the infant.

Characteristics	Total	HT Group	SP Group	*p*-Value ^2^	NMS	LMS	*p*-Value ^3^
EBM volume per feed (mL)	*n* = 168	*n* = 97	*n* = 71		*n* = 57	*n* = 7	
	120 [100, 150] ^1^	140 [120, 150]	110 [80, 150]	**<0.001**	120 [100, 150]	60 [47, 120]	**0.011**
EBM intake (mL/24 h)	*n* = 164	*n* = 95	*n* = 69		*n* = 58	*n* = 8	
	750 [600, 900]	800 [675, 900]	650 [500, 800]	**0.001**	750 [650, 820]	368 [300, 475]	**<0.001**
Commercial milk formula fed to the infant	*n* = 176	*n* = 98	*n* = 78		*n* = 59	*n* = 9	
	62 (35.2)	38 (38.8)	24 (30.8)	0.27	14 (23.7)	5 (55.6)	**0.036**
Infant age at formula introduction (months)	*n* = 62	*n* = 38	*n* = 24		*n* = 14	*n* = 5	
	0.5 [0.03, 2.0]	1.0 [0.03, 2.0]	0.03 [0.03, 1.03]	0.38	0.03 [0.03, 1.8]	0.03 [0.03, 0.03]	0.16
Formula volume per feed (mL)	*n* = 55	*n* = 36	*n* = 19		*n* = 13	*n* = 5	
	120 [100, 180]	150 [120, 250]	100 [60, 150]	**0.005**	120 [100, 160]	120 [65, 160]	0.83
Formula intake (mL/24 h)	*n* = 56	*n* = 36	*n* = 20		*n* = 13	*n* = 5	
	230 [150, 415]	215 [150, 400]	285 [120, 465]	0.79	170 [110, 200]	600 [270, 775]	**<0.001**
Total milk intake (mL/24 h) ^4^	*n* = 162	*n* = 94	*n* = 68		*n* = 57	*n* = 8	
	810 [700, 1000]	900 [720, 1001]	750 [600, 900]	**0.001**	800 [675, 900]	850 [364, 1118]	0.70
Solid foods fed to the infant	*n* = 176	*n* = 98	*n* = 78		*n* = 59	*n* = 8	
	56 (31.8)	35 (35.7)	21 (26.9)	0.21	3 (5.1)	1 (12.5)	0.41
Infant age at solid food introduction (months)	*n* = 56	*n* = 35	*n* = 21		*n* = 3	*n* = 1	
	6.0 [5.0, 6.0]	5.5 [5.0, 6.0]	6.0 [6.0, 6.0]	**0.005**	6.0 [5.5, 6.0]	4.0 [4.0, 4.0]	NA

^1^ Data are *n* (%) or median [Q1, Q3]. ^2^ *p*-value indicates significant difference between HT (healthy term group—women with term infants who did not have a neonatal unit admission) and SP (sick/preterm group—women with infants who were born preterm (<37 weeks birth gestation) and/or had a neonatal unit admission) groups, ^3^ *p*-value indicates significant difference between LMS and NMS groups using unpaired Student’s *t*-test or Chi-square test where appropriate. Bold font indicates a significant difference. ^4^ Sum of EBM volume and formula volume fed in 24 h. EBM, expressed breast milk; LMS, low milk supply (<600 mL/24 h) between 1.0 and 6.0 months postpartum; NA, not applicable; NMS, normal milk supply between 1.0 and 6.0 months postpartum.

**Table 5:** Exclusively pumping women’s pumping equipment details.

Characteristics	Total	HT Group	SP Group	*p*-Value ^2^
How is breast milk expressed? ^3^	*n* = 190	*n* = 104	*n* = 86	
Hospital grade electric breast pump	143 (75.3) ^1^	72 (69.2)	71 (82.6)	**0.034**
Wearable breast pump	118 (62.1)	70 (67.3)	48 (55.8)	0.10
Personal electric breast pump	80 (42.1)	40 (38.5)	40 (46.5)	0.26
Manual breast pump	39 (20.5)	25 (24.0)	14 (16.3)	0.19
Hand expression	35 (18.4)	13 (12.5)	22 (25.6)	**0.021**
Using more than one pump	*n* = 188	*n* = 102	*n* = 86	
Yes	155 (82.4)	86 (82.7)	69 (82.1)	0.46
Number of pumps used	1.0 [1.0, 2.0]	1.0 [1.0, 2.0]	1.0 [1.0, 1.0]	0.14
Prevalence of pump type used	*n* = 188	*n* = 102	*n* = 86	0.41
Hospital grade electric breast pump	89 (47.3)	43 (42.2)	46 (53.5)	
Wearable breast pump	51 (27.1)	33 (32.4)	18 (20.9)	
Personal electric breast pump	42 (22.3)	23 (22.5)	19 (22.1)	
Manual breast pump	4 (2.1)	2 (2.0)	2 (2.3)	
Other	2 (1.1)	1 (1.0)	1 (1.2)	
Vacuum setting used	*n* = 184	*n* = 100	*n* = 84	1.00
Low	19 (10.3)	10 (10.0)	9 (10.7)	
Medium	108 (58.7)	59 (59.0)	49 (58.3)	
High	46 (25.0)	25 (25.0)	21 (25.0)	
Other	7 (3.8)	4 (4.0)	3 (3.6)	
Cannot change	4 (2.2)	2 (2.0)	2 (2.4)	
Breast pump shield size used (mm)	*n* = 180	*n* = 100	*n* = 80	
	21 [19, 24]	21 [19, 24]	23 [19, 24]	0.088
Pumping bra used	*n* = 155	*n* = 79	*n* = 76	
Yes	106 (68.4)	57 (72.2)	49 (64.5)	0.086

^1^ Data are *n* (%) or median [Q1, Q3]. ^2^ *p*-value indicates significant difference between HT (healthy term group—women with term infants who did not have a neonatal unit admission) and SP (sick/preterm group—women with infants who were born preterm (<37 weeks birth gestation) and/or had a neonatal unit admission) groups using unpaired Student’s *t*-test, Chi-square or Fisher’s exact test where appropriate; bold font indicates a significant difference. ^3^ Sum of percentages > 100%, as participants could select more than one response.

**Table 6:** Exclusively pumping women’s breast pump selection details.

Characteristics	Total	HT Group	SP Group	*p*-Value ^2^
How was breast pump acquired	*n* = 188	*n* = 102	*n* = 86	0.94
Purchased after birth	100 (53.2) ^1^	55 (53.9)	45 (52.3)	
Purchased during pregnancy	48 (25.5)	26 (25.5)	22 (25.6)	
Borrowed from friend	18 (9.6)	10 (9.8)	8 (9.3)	
Hired	12 (6.4)	5 (4.9)	7 (8.1)	
Gifted	10 (5.3)	6 (5.9)	4 (4.7)	
How did you decide what pump to acquire? ^3^	*n* = 190	*n* = 104	*n* = 86	
Online reviews	79 (41.6)	48 (46.2)	31 (36.1)	0.16
Friends/family	69 (36.3)	47 (45.2)	22 (25.6)	**0.005**
Social media	68 (35.8)	45 (43.3)	23 (26.7)	**0.018**
Lactation consultant	52 (27.4)	26 (25.0)	26 (30.2)	0.42
Online forum	37 (19.5)	24 (23.1)	13 (15.1)	0.17
Midwife	23 (12.1)	9 (8.7)	14 (16.3)	0.12
Hospital/NICU pump	6 (3.2)	0 (0.0)	6 (7.0)	**0.008**
Other	11 (5.8)	2 (1.9)	9 (10.5)	**0.025**
Most important aspects of breast pump ^3^	*n* = 190	*n* = 104	*n* = 86	
Removes good volumes of milk	161 (84.7)	88 (84.6)	73 (84.9)	0.96
Mobility	119 (62.6)	74 (71.2)	45 (52.3)	**0.008**
Comfortable	117 (61.6)	67 (64.4)	50 (58.1)	0.38
Ease of transport	84 (44.2)	51 (49.0)	33 (38.4)	0.14
Affordable	75 (39.5)	43 (41.3)	32 (37.2)	0.56
Removes milk fast	68 (35.8)	37 (35.6)	31 (36.0)	0.95
Quiet	49 (25.8)	29 (27.9)	20 (23.3)	0.47
Other	3 (1.6)	2 (1.9)	1 (1.2)	1.00

^1^ Data are n (%). ^2^ *p*-value indicates significant difference between HT (healthy term group—women with term infants who did not have a neonatal unit admission) and SP (sick/preterm group—women with infants who were born preterm (<37 weeks birth gestation) and/or had a neonatal unit admission) groups using Chi-square or Fisher’s exact test where appropriate; bold font indicates a significant difference. ^3^ Sum of percentages > 100%, as participants could select more than one response. NICU, neonatal intensive care unit.

**Table 7:** Exclusively pumping women’s pumping kit storage and cleaning details.

Characteristics	Total	HT Group	SP Group	*p*-Value ^2^
Where is pumping kit stored?	*n* = 174	*n* = 93	*n* = 81	**0.006**
Refrigerator	101 (58.1) ^1^	65 (69.9)	36 (44.5)	**<0.001**
Bench at room temperature	43 (24.7)	16 (17.2)	27 (33.3)	**0.010**
Container at room temperature	30 (17.2)	12 (12.9)	18 (22.2)	0.11
Wearable left in bra	0 (0.0)	0 (0.0)	0 (0.0)	1.00
How is pumping kit cleaned? ^3^	*n* = 188	*n* = 102	*n* = 86	
Soap and water	160 (85.1)	89 (87.3)	71 (82.6)	0.37
Steam sterilizer	79 (42.0)	47 (46.1)	32 (37.2)	0.22
Rinse with water	47 (25.0)	26 (25.5)	21 (24.4)	0.87
Microwave	39 (20.7)	15 (14.7)	24 (27.9)	**0.026**
Boil on stove	25 (13.3)	16 (15.7)	9 (10.5)	0.39
UV sterilizer	25 (13.3)	12 (11.8)	13 (15.1)	0.50
Sterilizing solution	22 (11.7)	13 (12.7)	9 (10.5)	0.66
Dishwasher	13 (6.9)	11 (10.8)	2 (2.3)	**0.040**
Other	3 (1.6)	3 (2.9)	0 (0.0)	0.25
How often is pumping kit cleaned?	*n* = 190	*n* = 104	*n* = 86	**<0.001**
After each use	95 (50.0)	35 (33.6)	60 (69.8)	**<0.001**
Once per day	85 (44.7)	63 (60.6)	22 (25.5)	**<0.001**
Other	10 (5.3)	6 (5.8)	4 (4.7)	1.00
Who cleans pumping kit?	*n* = 176	*n* = 98	*n* = 78	0.47
Mostly me	117 (66.4)	67 (68.4)	50 (64.1)	
Shared with partner	55 (31.3)	30 (30.6)	25 (32.1)	
Mostly partner	4 (2.3)	1 (1.0)	3 (3.8)	

^1^ Data are *n* (%). ^2^ *p*-value indicates significant difference between HT (healthy term group—women with term infants who did not have a neonatal unit admission) and SP (sick/preterm group—women with infants who were born preterm (<37 weeks birth gestation) and/or had a neonatal unit admission) groups using Chi-square or Fisher’s exact test where appropriate; bold font indicates a significant difference. ^3^ Sum of percentages > 100%, as participants could select more than one response. UV, ultraviolet.

**Table 8:** Expressed breast milk storage details and cessation of exclusive pumping and expressed breast milk feeding.

Characteristics	Total	HT Group	SP Group	*p*-Value ^2^
Average EBM surplus (mL/24 h) ^3^		*n* = 159	*n* = 91	*n* = 68
	100 [0, 350] ^1^	100 [0, 250]	165 [0, 408]	0.10
Do you have EBM stored in a freezer?	*n* = 176	*n* = 98	*n* = 78	0.53
Yes	135 (76.7)	78 (79.6)	57 (73.1)	
No	18 (10.2)	8 (8.2)	10 (12.8)	
Had previously	23 (13.1)	12 (12.2)	11 (14.1)	
Freezer type	*n* = 135	*n* = 78	*n* = 57	**0.047**
Chest freezer	72 (53.3)	36 (46.2)	36 (63.2)	0.051
Fridge freezer	60 (44.4)	41 (52.6)	19 (33.3)	**0.026**
Other	3 (2.2)	1 (1.3)	2 (3.5)	0.57
Plans for frozen EBM ^4^	*n* = 176	*n* = 98	*n* = 78	
To feed infant, when EP ceased	105 (59.7)	61 (62.2)	44 (56.4)	0.43
Supplement fresh EBM	41 (23.3)	25 (25.5)	16 (20.5)	0.44
Donate	28 (15.9)	15 (15.3)	13 (16.7)	0.81
Unsure	18 (10.2)	12 (12.2)	6 (7.7)	0.45
Other	11 (6.3)	7 (7.1)	4 (5.1)	0.76
Current amount of frozen EBM (L)	*n* = 124	*n* = 74	*n* = 50	
	5.0 [2.0, 15.0]	5.0 [1.5, 15.0]	5.0 [2.0, 16.3]	0.59
Maximum storage time for frozen EBM (months)	*n* = 130	*n* = 77	*n* = 53	
	6.0 [3.0, 10.0]	6.0 [3.0, 10.5]	6.0 [3.0, 9.0]	0.85
Expected infant age when EP will cease (months)	*n* = 168	*n* = 95	*n* = 73	
	12.0 [7.0, 12.0]	12.0 [7.0, 12.0]	12.0 [7.5, 14.5]	0.068
Expected infant age when EBM feeding will cease (months)	*n* = 147	*n* = 85	*n* = 62	
	12.0 [9.3, 17.5]	12.0 [9.0, 14.0]	12.0 [10.0, 18.0]	0.061

^1^ Data are *n* (%) or median [Q1, Q3]. ^2^ *p*-value indicates significant difference between HT (healthy term group—women with term infants who did not have a neonatal unit admission) and SP (sick/preterm group—women with infants who were born preterm (<37 weeks birth gestation) and/or had a neonatal unit admission) groups using unpaired Student’s *t*-test, Chi-square or Fisher’s exact test where appropriate; bold font indicates a significant difference. ^3^ EBM surplus calculated as a difference between average EBM pumped in 24 h and average infant EBM intake/24 h. ^4^ Sum of percentages > 100%, as participants could select more than one response. EBM, expressed breast milk; EP, exclusive pumping.

**Table 9:** Returning to work and exclusive pumping for future children.

Characteristics	Total	HT Group	SP Group	*p*-Value ^2^
How soon after birth will you return to work?	*n* = 171	*n* = 96	*n* = 75	**0.017**
<4 weeks	6 (3.5) ^1^	3 (3.1)	3 (4.0)	1.00
1–3 months	6 (3.5)	5 (5.2)	1 (1.3)	0.23
3–6 months	13 (7.6)	11 (11.5)	2 (2.7)	**0.040**
6 months–1 year	70 (40.9)	45 (46.9)	25 (33.3)	0.065
1 year>	61 (35.7)	26 (27.1)	35 (46.7)	**0.016**
Not returning to work	15 (8.5)	6 (6.3)	9 (12.0)	0.28
Does returning to work influence EP?	*n* = 137	*n* = 85	*n* = 52	0.31
Very strongly	15 (11.0)	11 (12.9)	4 (7.7)	
Strongly	13 (9.5)	8 (9.4)	5 (9.6)	
Moderate	11 (8.0)	5 (5.9)	6 (11.5)	
Slightly	21 (15.3)	16 (18.8)	5 (9.6)	
Not at all	77 (56.2)	45 (52.9)	32 (61.5)	
Would you EP for future children?	*n* = 131	*n* = 69	*n* = 62	0.12
Yes, if infant could not be directly breastfed	90 (68.7)	43 (62.3)	47 (75.8)	
Yes, preferred method	14 (10.7)	7 (10.1)	7 (11.3)	
No	27 (20.6)	19 (27.5)	8 (12.9)	

^1^ Data are *n* (%). ^2^ *p*-value indicates significant difference between HT (healthy term group—women with term infants who did not have a neonatal unit admission) and SP (sick/preterm group—women with infants who were born preterm (<37 weeks birth gestation) and/or had a neonatal unit admission) groups using Chi-square or Fisher’s exact test where appropriate; bold font indicates a significant difference. EP, exclusive pumping.

## Data Availability

The data presented in this study are available on request from the corresponding author due to ethical restrictions.

## Abbreviations

The following abbreviations are used in this manuscript:

EBM	Expressed breast milk
EP	Exclusive pumping
GDM	Gestational diabetes mellitus
HT	Healthy term
LMS	Low milk supply
MP	Milk production
NICU	Neonatal intensive care unit
NMS	Normal milk supply
REDCap	Research Electronic Data Capture
SP	Sick/preterm
WHO	World Health Organization

## Appendix A

**Table A1:** Aspects of pump design which need improving.

Aspect of Pump Design That Needs Improving ^2^	Total *n* = 144
Price	25 (13.6) ^1^
More flange size options	21 (11.4)
Noise	12 (6.5)
Mobility whilst wearing	11 (6.0)
Size	11 (6.0)
Battery	10 (5.4)
Transport	9 (4.9)
Comfort	9 (4.9)
Wearable	8 (4.3)
Hands-free options	7 (3.8)
Ease of cleaning	6 (3.3)
Replacement parts	6 (3.3)
Vacuum strength	5 (2.7)
Large breast improvement	4 (2.2)
Reducing spillage	4 (2.2)
Efficiency	3 (1.6)
Larger milk collection bottles	2 (1.1)

^1^ Data are *n* (%). ^2^ Sum of percentages > 100%, as participants could select more than one response.
